# Cross-cultural adaptation and psychometric validation of the World Health Organization quality of life-old module (WHOQOL-OLD) for Persian-speaking populations

**DOI:** 10.1186/s12955-020-01316-0

**Published:** 2020-03-11

**Authors:** Hassan Rezaeipandari, Mohammad Ali Morowatisharifabad, Asghar Mohammadpoorasl, Abdolreza Shaghaghi

**Affiliations:** 1 Department of Health Education and Promotion, Faculty of Health, Tabriz University of Medical Sciences, Tabriz, Iran; 2 Department of Aging Health, School of Public Health, Shahid Sadoughi University of Medical Sciences, Yazd, Iran; 3 Health and Environment Research Center, Tabriz University of Medical Sciences, Tabriz, Iran

**Keywords:** Quality of life, Older adult, WHOQOL-OLD, Psychometric properties

## Abstract

**Background:**

Reliable quality of life assessment is important for identification of health problems, evaluation of health interventions and planning of optimal health policies and care packages. Due to lack of a psychometrically robust measurement tool for quality of life appraisal among the Iranian older population, this study was aimed to investigate psychometric properties of the Persian version of the World Health Organization quality of life-old module (WHOQOL-OLD-P) for use on the Iranian and other Persian-speaking aged populations.

**Methods:**

The standard translate/back-translate procedure was applied to convert the English version of the WHOQOL-OLD into Persian. The face and content validities were assessed by a panel of experts including 15 specialists in geriatrics and allied fields. The Cronbach’s alpha and intra-class correlation (ICC) coefficients were estimated to assess internal validity and reliability of the translated version. Factorial structure of the WHOQOL-OLD-P was also tested using confirmatory factor analyses in a sample of 400 Persian-speaking older adults (aged 60 years of old and above) residing in the city of Yazd, the capital city of Yazd province, center of Iran.

**Results:**

The internal consistency and reliability indices of the WHOQOL-OLD-P were in the vicinity of acceptable range (Cronbach’s alpha: 0.65–0.82 and ICC: 0.90–0.98). The confirmatory factor analysis outputs confirmed the six-factor solution of the WHOQOL-OLD-P (RMSEA = 0.04, CFI = 0.94, TLI = 0.93, SRMR = 0.06).

**Conclusion:**

The study findings support validity and reliability of the WHOQOL-OLD-P for use on Iranian and possibly other Persian-speaking older populations. Further cross-cultural and comparative multinational studies are recommended to provide more vigorous evidence about feasibility and acceptability of the translated tool in diverse and multicultural Persian-speaking communities.

## Background

Quality of life (QOL) assessment is considered by health scholars as an important requirement for evidence based health care decision making and policy formulation for ageing population of the world. Therefore, QOL assessment is a part of routine health care packages in many countries to ensure effectiveness of health care or innovative interventions that targeted aged populations [1, 2].

The ratio of aged to overall population of Iran as a transitional country is rapidly increasing and based on the 2016 demographic census’s results aged people constituted about 9.3% of the country’s population [3]. Current pace of notable growth in the global ageing population demands an extensive source of health and social care. Therefore, without an intensive plan of action to address maintenance of aged people’s health, the anticipated costs could be unaffordable for many countries, families and individuals [4].

QOL according to the World Health Organization (WHO)‘s definition is the self-perception of individuals’ current standpoint position in life with respect to what goals, expectations, standards, priorities, culture and value system they might have in their living ambience [5]. Thus, the concept of QOL could reflect many aspects of life [6] and its improvement might pose substantial effects on elder people’s life. In consequence; QOL measurement was endorsed as a reliable priority index to provide information about integrity and competence of a health care system in responding to the transitional needs of an aging population.

Application of a reliable and explicit tool to measure QOL of elder people is an imperative prerequisite in health systems and also for conduction of robust research projects [7]. However; few instruments such as CASP 19 [8] and OPQOL-brief [9] have been introduced for use sparingly on a verity of methodological and applied grounds. The existent generic and disease-specific tools for QOL measurement, usually have limitations for application on aged people populations due to empirical evidence of measurement bias and little standardization [10].

The WHOQOL-OLD as an explicit measurement instrument for QOL assessment was introduced by the WHO [11] and has been validated for application in different languages and socio-cultural circumstances [11–19] but not translated for use among Persian-speaking elder adults.

Preeminent advantage of the WHOQOL-OLD relative to other QOL assessment tools is its development through an extensive international cooperation [8] that makes its cross-cultural application and comparison of the results remissible. Being an old-age specific measure, not having other nonexclusive tools’ impediments (e.g. being developed inherently for general or young age populations and problems arising due to the format of administration, consistency of responses) and its applicability for impact assessment of the public and health policies on elder people’s quality of life are other superiority aspects of the WHOQOL-OLD. The instrument therefore; can be considered as a unique data collection tool that retain the required configuration for ascertainment of best investment areas to enhance quality of life in aged populations [8–11].

Main aim of this study was to translate and psychometrically appraise properties of the Persian version of the WHOQOL-OLD to provide a robust measurement instrument for application in akin studies of QOL on aged Persian-speaking populations in the Middle East region and other neighborhood countries. Thus, cross-cultural and international comparisons of the findings from studies in different populations will be applicable.

## Materials and methods

### Participants

The study sample consisted of 400 older adults (200 Zoroastrian and 200 Muslim individuals) aged 60 years old and above who were registered in the two primary health care center in the city of Yazd, the capital city of Yazd province, center of Iran. The sample size was decided based on the recommended sample size (8 people per each of the instrument’s items) [20] for performing the confirmatory factor analyses. The invited aged people for participation in the study were 480 registred Zoroastrians (260) and Muslim (220) dwellers of them 400 consented to cooperate in the study (particiaption rate (83.33%).

The inclusion criteria were being permenant resident of the city of Yazd, having age equal to 60 or above, being diagnosed with at least one to three simulataneous chronic diseases, ability to speak and understand Persian language, not having considerable hearing loss to make interviews absurd and also not having a major cognitive problems which was assured based on the respondents’ Abbreviated Mental Test scores (AMT) [21]. The study exclusion criteria composed of not willing to continue participation after giving informed consent, progression of a chronic disease that make participation in the study medically inconsistent or development of a severe disability that make the respondent epistemically dependent on others.

### Translation procedure

Standard translation and back translation procedures were followed after formal permission from the WHO Quality of Life (WHOQOL) assessment group to prepare a preliminary draft of the WHOQOL-OLD-P [22, 23]. The original WHOQOL-OLD was first translated into the Persian language by the two professional translators, one of them was familiar with the study subject. At the next stage the translated draft was back translated into the English language and this back-translated version was compared side by side to the original WHOQOL-OLD in terms of clarity, common language use, conceptual equalization and quality of the overall translation. Any inconsistencies between the original and the translated version was resolved at this stage and the final Persian version was administered to a group of representative older people (*n* = 10) unfamiliar with the topic, to ask for their comments about vague items or phrases, format of the questions and response items. All the feedbacks were considered to ensure rapport and having an exactly equivalent translated version to the original WHOQOL-OLD.

### Content validity appraisal

A panel of experts consisting 15 gerontologists, geriatricians, health education and promotion specialists, psychologists and nursing specialists were contacted for their comments about the simplicity, grammatical soundness, clarity and understandability of the WHOQOL-OLD-P. The Lawshe’s Content validity index (CVI) and content validity ratio (CVR) were estimated based on the panelists feedbacks and values of CVI ≥ 0.8 [24] and CVR ≥ 0.49 [25] were deemed acceptable. To ensure face and qualitative content validity of the WHOQOL-OLD-P it was also pilot tested on the 28 older adults for their comments about understandability of the questions and response items however; no important issue was mentioned.

### Reliability assessment

The Cronbach’s alpha coefficient of internal consistency and the Intra-Class Correlation (ICC) coefficient of reliability were calculated to assess performance of the WHOQOL-OLD-P and reproducibility of the data collection process.

### Instrument

#### WHOQOL-OLD

The WHOQOL-OLD is a multidimensional measure of quality of life in older adults that consist 24 items in six domains [11]. The items’ responses are scored on a Likert type scale ranging from 1 to 5 and a higher total score represents better quality of life. The six subscales of the instrument include the respondent’s perception about his/her sensory abilities, autonomy, lifetime activities, social participation, intimacy with others, death and dying [11].

### Data collection

Two trained and qualified interviewers were hired to collect data in face-to-face interviews from the Zoroastrian and their socio-economically matched aged Muslim counterparts who had been registered in the two purposefully selected urban health care centers.

### Data analysis

The skewness and kurtosis of the collected data were tested for normal distribution, mean and standard deviation of the WHOQOL-OLD-P’s scores were estimated consequently [26]. The ceiling and floor effects were also checked for detection of imbalance in the percentage of the study participants (greater than 20%) with low and high levels of WHOQOL-OLD-P’s scores and to assess ability of the instrument in discriminating the respondents across the high and low categories of quality of life [10]. The internal consistency reliability coefficient of Cronbach’s Alpha and the intra-class correlation coefficient (ICC) to test the WHOQOL-OLD-P stability over time were estimated and values above 0.7 were deemed satisfactory [27, 28].

To verify compatibility of the study findings with the factor structure reported for the original WHOQOL-OLD module, confirmatory factor analysis (CFA) was performed and the CFA fit indices including Chi-squared/df < 5.00, Standardized Root Mean Square Residual (SRMR) < 0.08, Comparative Fit Index (CFI), and Tucker Lewis index (TLI) > 0.90 were deemed to be acceptable [29]. The IBM SPSS version 24 (IBM Corporation, Software Group, Armonk, NY, USA; 2016) [30], and STATA version 14 (Stata Corporation, College Station, TX: LP; 2015) [31] software were used for data analysis purposes.

## Results

### Participants characteristics

Mean age of the study participants was 70.63 ± 8 and their mean WHOQOL-OLD-P’s scores was 77.72 ± 10.41. Other characteristics of the respondents were presented in Table 1.

**Table 1:** Demographics characteristics of the study participants (*n* = 400) in the cross-cultural adaptation and psychometric validation of the World Health Organization quality of life- old module (WHOQOL-OLD) for Persian-speaking populations

Variables	n (%)
**Gender**	
Male	167 (41.8%)
Female	233 (58.2%)
**Marital status**	
Married	288 (72.0%)
Non-married	112 (28.0%)
**Educational level**	
Illiterate	71 (17.7%)
Primary school	126 (31.5%)
Secondary school	38 (9.5%)
High school diploma	23 (5.8%)
Academic undergraduate degree	104 (26.0%)
Academic postgraduate degree	38 (9.5%)
**Retirement status**	
Retired	166 (41.5%)
Not retired	234 (58.5%)
**Living status**	
With spouse	204 (51.0%)
With spouse & children	80 (20.0%)
With only unmarried children	14 (3.5%)
With only married children	40 (10.0%)
With other relatives/friends	8 (2.0%)
Living alone	54 (13.5%)
**Morbidities**	
Hypertension	250 (62.5%)
Cardiovascular diseases	96 (24.0%)
Cancer	16 (4.0%)
Pulmonary diseases	48 (12.0%)
Diabetes	161 (40.3%)
Osteoporosis	107 (26.8%)
Arthritis	237 (59.3%)
Hyperlipidemia	177 (44.3%)

### Feasibility

The study participants’ overall and subscales’ scores of the WHOQOL-OLD-P along with the test-retest reliability and internal consistency coefficients were tabulated in Table 2. The outputs of ceiling and floor effects’ analysis for overall and subscales of the instrument were also presented in this table. As indicated the estimated values are in the range of acceptable range.

**Table 2:** The WHOQOL-OLD-P overall and subscales’ scores of the study respondents in the cross-cultural adaptation and psychometric validation of the World Health Organization quality of life- old module (WHOQOL-OLD) for Persian- speaking populations

WHOQOL-OLD-P subscales	Minimum score	Maximum score	Mean score	SD	Skewness	Kurtosis	Floor (%)	Ceiling (%)	Cronbach’s α	ICC
**Sensory abilities**	4	20	13.98	3.16	−0.39	−0.09	0.5	1.3	0.83	0.95
**Autonomy**	4	20	12.67	2.63	−0.18	1.03	0.8	1.3	0.75	0.95
**Death and dying**	4	20	10.89	4.10	0.34	−0.48	8	3.8	0.83	0.98
**Social participation**	5	20	13.45	2.54	−0.38	0.68	0.3	1.5	0.73	0.98
**Past, present and future activities**	6	20	13.52	2.38	−0.09	0.67	0.5	1.3	0.80	0.95
**Intimacy**	4	20	13.20	2.93	−0.33	0.45	1.3	1.8	0.78	0.90
**Total**	44	114	77.72	10.44	−0.22	0.87	0.3	0.3	0.82	0.91

### Qualitative content and face validity appraisal

The WHOQOL-OLD-P was sent to a panel of experts for their feedbacks about the instrument items’ conceptual, cultural and linguistic appositeness. Based on the panelists’ given scores to the listed items’ attributes on a Likert type scale the CVI and CVR were estimated and the values were in the range of acceptable variability (0.88 and 0.86 respectively).

### Quantitative construct validity appraisal

The conducted CFA in a sample of 400 Muslim and Zoroastrian older adults yielded an acceptable model fit with the six factor solution that was similar to the endorsed model fit for the original WHOQOL-OLD (χ^2^/df = 1.92, *P* < 0.001), RMSEA = 0.04, CFI = 0.94, TLI = 0.93, SRMR = 0.06).

## Discussion

Main aim of this study was to verify psychometric properties of the WHOQOL-OLD-P for use on Persian-speaking older adults in Iran and probably other countries of the world. The study findings represented admissible reliability and construct validity of the instrument to be used for QOL assessment in research and conceivably in practice settings.

In the development of the original WHOQOL-OLD module, the interviewees’ scores were considered to exhibit floor/ceiling effects on the basis of the percentage of the respondents with maximum or minimum total scores that exceeds 20% [11]. The WHOQOL-OLD-P by this categorization, connoted no substantial floor and ceiling effects, indicative of its discriminant ability identical to the findings of the other WHOQOL-OLD cross-cultural validation studies [14, 17, 19, 32, 33]. However; in a number of previous studies a tendency towards ceiling effect [11, 34, 35] in measurement of well-being, life satisfaction and QOL was suggested due to probability of rampant positivity bias [8].

The estimated internal consistency measures of reliability (Cronbach’s alpha coefficient) for the total and subscales of the WHOQOL-OLD-P in the present study were in the acceptable range (> 0.7) [40] which is somehow consistent with the findings of other psychometric studies of the WHOQOL-OLD module on other socio-cultural contexts [11–17, 19, 36]. The only observed incongruity among the conducted validation studies were low estimated internal consistency measure of the Cronbach’s alpha for the “social participation” subscale in the Australasian study [37], for the autonomy subscale in the Spanish, German and Turkish studies [12, 17, 19] and for the “past, present and future activities” subscale in the Norwegian study [15].

The calculated ICC measure of reliability over time for both the total WHOQOL-OLD-P and its subscales’ scores were in the vicinity of acceptable range [28] and similar to the findings of two other validation studies of the WHOQOL-OLD [8, 16].

The identified 6-factor solution that best fitted the study data was congruent with the reported priori factor structure of the original scale [11] and in the validation study of the Dutch version of the WHOQOL-OLD [38], however; the hypothesized model structure of the instrument in other studies [12, 13, 15, 16, 19, 32] were incompatible.

Regarding the obtained results in this validation study, it can be concluded that the WHOQOL-OLD-P’s application is feasible since no problem encountered in the data collection stage and a relatively limited time frame (6–8 min for older adults with academic education and 10–13 min for the rest of interviewees) was required to complete the scale on every individual participant.

### Study limitations

The study sample in this research consisted of elder people with at least one to three diagnosed simultaneous chronic diseases who had been registered in the two primary health care centers in the city of Yazd, the capital city of Yazd province, central part of Iran. The purposively selected health care centers and therefore, non-probabilistic sample that was selected for inclusion might be limited representativeness of the study participants and restraint extrapolation and generalizability of the findings. Comparison of the within-subjects perceived QOL measures and association of the factors such as religion, age, gender, level of education and overall health status with the estimated QOL values were not speculated in this study. Therefore; interim data collection and analysis in future corresponding studies could add further detail and explanation about precipitating factors of QOL.

Iran is a multi-cultural, multi-religious and multi-ethnic country and the study findings might not be generalizable to elder people residing in the country’s other provinces and geographical areas due to socio-cultural diversities.

There is also possibility of the reporting bias based on the respondents’ willingness and ability to provide accurate responses especially concerning the length of the WHOQOL-OLD-P, although the concern was attempted to be mitigated by emphasis on the anonymity of the participants and allocation of proper time for interview.

Elder people who suffer from multiple chronic diseases are more likely to develop depression and therefore; tend to report lower quality of life [38]. Such an impediment must be taken into account in interpretation of the study data.

Multidimensional nature of QOL and the effects posed by certain underlying factors warrant further research to examine how these circumstances might relate to self-reported quality of life among older people. QOL assessment by use of measures that actually highlight specific domains e.g. physical, emotional and social functioning or over emphasis on health-related aspect of daily life experiences may also cause bias especially in measurement of elder population’s QOL [39]. Aged people themselves, might have a priority need or interest that could potentially be reflected in their responses to the items of QOL assessment instruments. The judgmental approach (which entails comparison between the respondents’ state in different time frames e.g. present to past) which is salient generally in health related quality of life assessment tools could also further contribute to ambiguity in reliable determination of QOL. A number of other baseline socio-demographic factors e.g. gender, being divorced, unemployed, migrant and bereaved might also have same or even higher impact on several dimensions of perceived QOL in aged populations [39]. All these facets warrants interpretation of the QOL data with caution.

Introduction of a valid and reliable instrument for measurement of quality of life among Iranian and other Persian-speaking elder adults was the primary objective of this study. Taking into account all above mentioned limitations, the WHOQOL-OLD-P application could be recommended as an explicit instrument for measurement of perceived QOL as a proxy measure of aged people’s overall health in the Iranian and other Persian-speaking elder populations across the world. The WHOQOL-OLD-P application could enhance reliability of QOL assessment by a wide range of healthcare providers (HCPs) (e.g. nurses, psychologists, geriatricians) in primary health care centers, secondary and tertiary care providing settings (e.g. nursing homes, day care centers and hospitals). The instrument can be utilized in routine care provision activities or in assessment of influences a specific health condition or morbidity and also subsequent restorative therapeutic intervention might have on perceived overall QOL among the aged people. Other social care providing organizations could also use the WHOQOL-OLD-P for estimation of impacts their policies, services or targeted interventions might have on elder people within the Persian-speaking countries.

## Conclusions

The WHOQOL-OLD module was translated into Persian in this study and psychometrically tested for possible application in the research and practice settings within the Iranian context. The translated version indicated robust internal consistency, reliability and construct validity. Due to growing number of aged population in Iran presence of sound data collection tool for QOL assessment will add to the accuracy of scientific knowledge and intervention modalities that target elder people’s quality of life in different socio-cultural and practice settings. The WHOQOL-OLD–P utilization in future studies will also make comparison of cross-region and cross-border health status of elder people attainable and desirable.

## Data Availability

The datasets used and/or analyzed during the current study are available from the corresponding author on reasonable request.

## Abbreviations

QOL: Quality of life
WHO: World Health Organization
WHOQOL-OLD: World Health Organization Quality of life-old module
AMTS: Abbreviated Mental Test Score
WHOQOL-OLD-P: World Health Organization Quality of life-old module for Persian-speaking population
CVI: Content validity index
CVR: Content validity ratio
ICC: Intra-Class Correlation
CFA: Confirmatory factor analysis
RMSEA: Root Mean Square Error of Approximation
SRMR: Standardized Root Mean Square Residual
CFI: Comparative Fit Index
TLI: Tucker Lewis index
HCPs: Healthcare Providers
